# Physical Activity and Associated Factors among Brazilian Adult Inmates: A Cross-Sectional Study

**DOI:** 10.3390/ijerph21060748

**Published:** 2024-06-07

**Authors:** Wanessa Cristina Baccon, Carlos Laranjeira, Priscila Garcia Marques, Carla Franciele Höring, Adriana Martins Gallo, Juliane Pagliari Araujo, Francielle Renata Danielli Martins Marques, Lígia Carreira, Maria Aparecida Salci

**Affiliations:** 1 Departamento de Pós-Graduação em Enfermagem, Universidade Estadual de Maringá, Avenida Colombo, 5790-Campus Universitário, Maringá 87020-900, PR, Brazil; wanessa.baccon@gmail.com (W.C.B.); adriana.gallo@ifpr.edu.br (A.M.G.); franrenata.martins@gmail.com (F.R.D.M.M.); ligiacarreira.uem@gmail.com (L.C.); masalci@gmail.com (M.A.S.); 2 School of Health Sciences, Polytechnic University of Leiria, Campus 2, Morro do Lena, Alto do Vieiro, Apartado 4137, 2411-901 Leiria, Portugal; 3 Centre for Innovative Care and Health Technology (ciTechCare), Rua de Santo André-66-68, Campus 5, Polytechnic University of Leiria, 2410-541 Leiria, Portugal; 4 Comprehensive Health Research Centre (CHRC), University of Évora, 7000-801 Évora, Portugal; 5 Departamento de Educação Física, Universidade Estadual de Maringá, Avenida Colombo, 5790-Campus Universitário, Maringá 87020-900, PR, Brazil; priscila.garcia.marques@gmail.com; 6 Departamento de Estatística, Universidade Estadual de Maringá, Avenida Colombo, 5790-Campus Universitário, Maringá 87020-900, PR, Brazil; cfhoring2@uem.br; 7 Departamento de Pós-Graduação em Enfermagem, Universidade Estadual de Londrina, Rodovia Celso Garcia Cid, PR-445, Km 380-Campus Universitário, Londrina 86057-970, PR, Brazil; juliane.pagliari@ifpr.edu.br

**Keywords:** inmates, physical activity, health, cross-sectional study, Brazil

## Abstract

Previous studies on health in prison facilities have determined that imprisonment has adverse effects on both physical and emotional well-being. Therefore, the introduction of public health measures is encouraged. This study aimed to (a) evaluate the levels of physical activity and the health condition of a sample of Brazilian prisoners and (b) determine the predictors of low physical activity. An observational and cross-sectional study was developed following the STROBE checklist. Data collection took place between June and November 2019 in a maximum-security Brazilian prison institution. This study’s final sample included 220 people selected through convenience sampling, of which 115 (53.2%) were aged 18 to 29 years, followed by 79 (36.6%) aged 30 to 44 years and 22 (10.2%) aged 45 to 59 years old. Overall, 64.3% of participants failed to meet the World Health Organization (WHO) recommendation for physical activity [at least 150–300 min of moderate-intensity or 75 min of vigorous-intensity aerobic physical activity per week]. The majority reported unhealthy food consumption (116; 53.7%). Regarding body mass index (BMI), 50.2% of individuals were classified as eutrophic, 38.1% were overweight and 11.6% were obese. Older age (AOR: 0.95; CI95%: 0.92–0.99; *p* = 0.01) and sitting time (AOR: 0.74; CI95%: 0.65–0.85; *p* < 0.01) were associated with low adherence to physical activity. Our results highlight the importance of practicing physical activity within the prison context and the need for institutional programs that promote regular physical activity.

## 1. Introduction

The imprisonment rate in Brazil has tripled in recent decades, from 104.7 to 367.9 per hundred thousand inhabitants. The country has the third largest prison population in the world, with a total of 644.316 inmates in 2023, behind the USA and China, which have the first and second positions, respectively [1,2]. Brazil is host to the unhealthiest prisons globally due to overcrowding [3], poor diet, lack of physical activities, violence, aggression, and lack of privacy [4]. Evidence indicates that a significant proportion of inmates suffer from subpar health or worse health conditions than the general population [5,6]. The rate of chronic medical conditions (including diabetes mellitus, hypertension, asthma, arthritis, or HIV) is higher than the general population [7].

Several factors elucidate the substandard health status among prisoners. The health of the prison population has never been seen as a priority, resulting in decades of neglect due to poor structural conditions in prisons and insufficient human resources [8]. The social vulnerability of prisoners intensifies the inequity felt by this population and their difficulty in accessing health services [9]. The experience of social confinement is a complex process that can threaten the individual’s physical and psychosocial integrity, as well as cause chronic stress and suffering [10,11]. Although veiled, there is social pressure concerning the services provided to this population, with a lack of assistance seen as punishment. At the same time, studying the health needs of people in prisons represents a challenge for researchers, as this population is heavily stigmatized by public opinion.

While the primary purpose of prisons is to foster the resocialization of inmates, a hostile and unhealthy prison environment can hinder the subsequent resocialization process [12,13]. Overcrowding and poor conditions in prisons have direct implications for health conditions and healthy lifestyles [14]. Available evidence considers that sporting activities play a significant role in resocialization and represent the most popular leisure activity among prisoners [15,16]. Physical exercise has a positive impact on mental well-being and protects against depression and anxiety, especially in individuals living in confined environments, as it causes biochemical and biophysical changes in the brain that can positively influence their mood [17].

Incarceration, combined with related health risk behaviors, is conducive to cardiovascular risk. Possible reasons for the association between incarceration and weight gain include “a sedentary lifestyle, unhealthy diet, forced smoking cessation, use of psychotropic medications, and high levels of stress” [18] (p. 1). Recent reviews suggest that closely monitored physical activity and exercise programs in prison enhance health indicators and decrease risk factors for cardiovascular illnesses [11,19,20]. Research from prison contexts also suggests the beneficial effects of sports on psychological and mental health, namely in stress and emotional regulation [21]. Considering that the prison population has a higher risk of hospitalization and death related to cardiovascular diseases when compared to the general population [22,23,24], it is necessary to develop health promotion strategies through nutritional monitoring, cardiovascular health assessment [10], and the promotion of physical activity [20].

In Brazil, few studies have evaluated physical activity and its associated factors in prison populations. One recent study reported that most inmates have high body mass indexes, identifying the need for health interventions [14]. Given the serious health problems of imprisoned populations, this study aims to (a) evaluate the levels of physical activity and the health condition of a sample of Brazilian prisoners and (b) determine the predictors of lower physical activity. The findings may contribute to implementing strategies to foster physical activity and prevent cardiovascular risk factors in prison environments.

## 2. Materials and Methods

### 2.1. Study Design

A quantitative, descriptive-correlational, and cross-sectional study was developed and reported according to the Strengthening the Reporting of Observational Studies in Epidemiology (STROBE) checklist [25].

### 2.2. Setting and Participants

This study was carried out in a maximum-security penal institution located in the northwest region of the state of Paraná, Brazil. Male adults incarcerated in this facility include those detained while awaiting trial or serving a sentence. Crimes pertaining to trafficking and drug usage, as well as crimes against property, are among the reasons for incarceration. The prison was designed to house 960 inmates, but, during data collection, there were close to 1200 inmates. Overcrowded jails, a challenge in Brazil, limit the appropriate indoor and outdoor facilities and the safe recreational areas that provide prisoners with sufficient opportunities to exploit the health benefits of physical activity. In this prison facility, inmates have access to outdoor space once or twice a week for approximately 6 h. Walking and football are the most common physical activities.

Using a convenience sampling method, individuals who met the following inclusion criteria were selected: (a) having been imprisoned on a provisional regimen (that is, those deprived of their liberty but not yet definitively sentenced); (b) period of imprisonment exceeding 30 days; and (c) presenting cognitive and intellectual capacity to participate in this study. All individuals diagnosed with mental medical conditions preventing participation, elderly inmates (i.e., over 60 years of age), or those absent from the prison or cell block on the days of data collection were excluded.

### 2.3. Data Collection

Data were collected in person from June to November 2019 by the main researcher (W.C.B.), who works as a nurse in the aforementioned prison establishment. During data collection, the nurse opted to wear civilian attire rather than a uniform, indicating that the data collection process was separate from and would not impact their path within the prison system. Among the 643 potential participants, 216 inmates agreed to participate in this study, resulting in a response rate of 33.6%.

### 2.4. Instrument

A questionnaire was constructed specifically for this study and pre-tested one week before the actual data collection on 10 inmates not included in the main study. Minor changes were made to the data collection tool for better readability. It included the following four parts:

(a) Personal and sociodemographic variables [age (years); race (white; black/African descent); years of study (<8 years; ≥8 years); have a partner (yes; no); has children (yes; no)];
(b) Living conditions before incarceration [housing condition (owned; rented); paid work (yes; no); family income in minimum wages (2019: BRL 998.00) (<1 MW; ≥1 MW)];
(c) Information about current incarceration [previous incarceration history (no; yes); time in prison (<1 year; ≥1 year); work in prison (no; yes); have had visitors in last month (no; yes)];
(d) Data on health and lifestyles: Blood pressure was evaluated by the main investigator (using a calibrated electronic device) and classified as optimal/normal (<129/84 mmHg) and limit/high (>130/85 mmHg) according to [26]. Body mass index (BMI) was categorized as eutrophic (18.5 < 25 kg/m^2^), overweight (≥25.0 <30 kg/m^2^), or obese (≥30 kg/m^2^). BMI was calculated using the respondent’s weight divided by the square of height (a researcher evaluated these anthropometric parameters). BMI was considered altered when ≥25 kg/m^2^ [27]. Sitting time (<12 and ≥12 h/day) has been identified as critical for several adverse outcomes, such as the risk of cardiovascular disease and all-cause mortality [28]. The weekly physical activity level, following the WHO recommendation [29], should be at least 150–300 min of moderate-intensity or 75 min of vigorous-intensity aerobic physical activity per week (no; yes). Food intake: healthy (consumption of fruits and vegetables ≥5 days a week, consumption of legumes ≥5 days a week, consumption of fish at least once a week, consumption of full-fat milk) and unhealthy (consumption of soft drinks ≥5 days a week and regular consumption of sweets ≥5 days a week) [30].

### 2.5. Data Analysis

The collected data were cleaned and exported to R Statistical Software (Version 4.2.0) [31]. Categorical variables were summarized using absolute and relative frequencies, mean, median, standard deviation, and range. The associations between these variables were verified using Fisher’s exact test and Pearson’s chi-square test. Data normality was checked using the Shapiro–Wilk test. Univariate and multiple binary logistic regression models were used to identify potential explanatory variables for inmates’ lower physical activity. The variables obtained in the bivariate analysis, with a *p*-value of less than 20% (*p* < 0.20), were inserted one by one into the multivariate model. The stepwise both-direction method was used to select variables and adjust the final models. The adequacy of these models was verified with the analysis of randomized quantile residuals and McFadden’s R^2^. Collinearity was tested with the variance inflation factor (VIF). Associations were estimated by calculating the odds ratio (OR), adopting the 95% confidence interval as a measure of precision. Significance was set at a *p* ≤ 0.05.

### 2.6. Ethical Issues

This study complied with national regulations and was approved by the Permanent Committee on Ethics and Research with Human Beings, under opinion 3.211.746, CAAE 08936619.4.0000.0104, on 20 March 2019. Participation was both anonymous and optional. All participants submitted written informed permission after receiving complete information about this study’s nature. Participants were explicitly notified that they could withdraw from this study at any time and could decline to answer any questions that caused them discomfort. During data collection, all information provided by participants was anonymized and maintained anonymously. There were no financial incentives for completing the poll.

## 3. Results

### 3.1. Sample Description

Prisoners had an average age of 30.3 ± 9.2 years, and most reported having a partner (70.8%) and children (65.3%) (Table 1). Overall, 64.3% of participants failed to meet the WHO recommendation for physical activity. Younger individuals, aged 18 to 29 years, showed greater involvement in physical activities compared to those in the older age groups (*p* = 0.034).

All participants were Brazilian, mostly black and mixed-race (61.6%). The majority of inmates were physically inactive (63.3%). Prisoners with fewer qualifications [<8 years: basic education] (60.6%) practiced more physical activity when compared to those with more years of education. Regarding living conditions before incarceration, around 60.2% had their own home. Most inmates reported working before incarceration (88.9%) and earning one or more national minimum wages (88%).

When analyzing the conditions of current incarceration, we observed that 73.6% of participants had a previous history of imprisonment, and most of them did not exercise regularly. Most had been in prison for less than one year and did not work inside the prison. Only 32.4% of participants did not receive family visits.

Based on the BMI classification, 50.2% of individuals were eutrophic, 38.1% were overweight, and 11.6% were obese. In each group, most prisoners reported having no regular physical activity. Concerning blood pressure, 79 participants had values above normal, of which 36.7% had no physical activity following WHO recommendations. The majority reported unhealthy food consumption (n = 116; 53.7%), with only 62.3% of inmates dedicating time to physical activity. As for sitting time, most reported daily values exceeding 12 h (73.5%). Non-adherence to physical activity was higher among participants with time sitting up to 12 h compared to inmates with values above 12 h (17.3% vs. 82.7%, *p* = 0.001).

### 3.2. Predictors of Lower Physical Activity

The univariate model revealed a significant correlation between the following factors and physical activity: age in years (OR: 0.96; 95% CI: 0.96–0.99; *p* = 0.008), sitting time per day (hours) (OR: 0.73; 95% CI: 0.64–0.83; *p* = 0.001), and unhealthy food intake (OR: 0.58; 95% CI: 0.33–1.02; *p* = 0.049).

After adjusting for potential confounders, the results revealed that age and sitting hours were associated with lower odds of physical activity. Compared to younger individuals in this study, older adults had a nearly onefold higher likelihood of not adhering to physical exercise (AOR: 0.95; 95% CI: 0.92–0.99; *p* = 0.010). Inmates with more sitting time were 0.7 times more likely to not practice physical activity than their counterparts (AOR: 0.74; 95% CI: 0.65–0.85; *p* < 0.001). Nevertheless, no statistically significant correlation was found between physical activity and the other variables examined in the multivariable logistic regression analysis (Table 2).

The final model explained 20.5% of the variance in physical activity based on the calculated amount of adjusted coefficient of determination (R2 adjusted).

## 4. Discussion

This study was one of the first Brazilian studies to evaluate the level of physical activity and health status of prisoners and to determine the predictors of low adherence to physical activity. The overall prevalence of inmates who failed to meet the WHO recommendation for physical activity was 64.3%, which is lower than that in reports from Israel, in which 82.37% of participants did not meet the recommended physical activity level [32]. Only two factors were significantly associated with lower physical activity in multivariate logistic regression analysis, namely, age and sitting time.

Similar results were observed in a Polish study, in which the ages of prisoners were significantly correlated with physical activity levels [33]. Inmates aged 18 and 29 and those over 40 reported low levels of physical activity, while those between 30 and 40 reported high or medium levels. No significant correlations were found between physical activity and eating habits, diet quality, or nutrition knowledge [33].

The beneficial impacts of physical activities on people’s lives are widely reported in the literature [34]. However, the discussion only rarely pertains to the prison population [12,33]. The worst outcomes are related to prisoners who do not practice regular physical activities. These are disproportionately affected by chronic diseases, with implications on physical and mental well-being [35]. The length of incarceration implies different contexts in the lives of prisoners, and the development of chronic diseases is common due to changes in the anthropometric parameters of this population [14]. Several reasons explain the high rates of sitting time among inmates. The typical prison environment makes it difficult for prisoners to carry out health-promoting behaviors like smoking cessation, healthy eating, or physical activity in the fresh air. Moreover, prison management often marginalizes health promotion and prisoners do not have access to adequate health care [36].

Access to regular physical activities is important to maintain and improve the health of prisoners [37]. In Brazil, the National Policy for Comprehensive Healthcare for Persons Deprived of Liberty recognizes the practice of sports in a prison environment as necessary to ensure the physical and mental health of inmates [38]. However, many penitentiaries do not foster this practice, promoting a culture of inactivity and idleness. At the same time, prisons in Brazil are still characterized as violent places with precarious and overcrowded facilities, which is reinforced by practices contrary to health promotion, such as the use of tobacco, alcohol, illicit drugs, and bad eating habits [12]. Our study indicates that although unhealthy eating did not remain in the final model, its relevance persists, suggesting that special attention should be given to eating habits and the practice of sports to promote the health and well-being of inmates in the Brazilian prison system.

Factors related to excess weight and obesity permeate behavioral and environmental issues as they are multifactorial. The weaknesses perceived by the prison population and the vulnerability to which they are exposed impact their health [39]. A Canadian retrospective cohort study that analyzed changes in relation to BMI and annual weight gain showed weight gain among inmates. Among the participants, habits such as physical activity, screen time, and sleep were self-reported, directly influencing the results. The association between physical inactivity and weight gain was highlighted, and inmates found it difficult to lose weight, even with high levels of daily physical activity (more than 60 min per day) during incarceration [40]. In line with our results, there was a similar proportion of overweight and normal-weight individuals, as also seen in a Polish study in which around 42% of respondents were overweight [33].

The scarcity of food and its lack of quality within the prison environment is a known reality in Brazilian prisons. In effect, there is a gap between the need to improve the nutritional standards of prisoners and the implementation of public actions and policies related to nutrition for vulnerable people and those experiencing social inequality [41]. Meals are usually standardized, and unlike in other countries, prisoners do not have the opportunity to buy snacks or meals in canteens, although they can receive food from family members [33].

During the COVID-19 pandemic, an increase in the consumption of ultra-processed foods was reported [42]. There are several factors involved, particularly related to the logistics of transporting and storing fresh products in hygienic conditions, which quickly became unfit for consumption. Thus, the consumption of processed foods increased due to the natural loss of perishable foods during the health crisis [9,43].

The Brazilian prison population has a higher prevalence of diseases than the general population. However, little is known regarding high blood pressure [44]. Incarceration is associated with an increased risk of high blood pressure and cardiovascular diseases and represents the main cause of death among prisoners [37]. In our research, 36.4% of the participants had changes in blood pressure, even when practicing physical activity. Once an inmate is prevented from fully exercising their rights, they are vulnerable to prison conditions that imply changes in their habits, in addition to difficulties in accessing healthcare that constrain the development of healthy habits [7,45].

Given our analysis, we strongly advocate a broader conceptualization of prison health and well-being and the development of clear criteria to promote health and examine the causes of poor health, not just the symptoms [46]. Evidence suggests an increased focus on how to avoid sedentary behavior [47], particularly when inmates are locked in cells for extended periods, and an increased emphasis on setbacks and relapse prevention to reflect the chaotic nature of participants’ lives and the competing demands on their attention.

### 4.1. Study Limitations

This study presents several limitations that deserve consideration in the prison context. Firstly, the adopted cross-sectional design implies that the observed results are unstable and do not provide causal evidence. Longitudinal designs could provide a more in-depth understanding of the factors investigated over time. Additionally, the results were only obtained from male inmates, who are mostly black with different lengths of incarceration and low levels of education, which limits the generalization of the results to the general population. Future studies can enrich their analyses by including participants of both sexes, adolescents, or inmates with different lengthy sentences enabling comparative analyses. This study was conducted solely on the Brazilian prison population, limiting its applicability to other geographic contexts. Another significant methodological limitation is the absence of a validated instrument to collect data on eating habits and the use of self-reported data. This may compromise the accuracy and reliability of the information collected. Because evidence suggests that mental health status predicts low physical activity [48], future research should combine multiple physical activity assessments and mental health screening tools to better evaluate the health status of prison inmates.

### 4.2. Implications for Practice

The assessment of physical activity levels in a prison population has implications for practice, research, and policies, prioritizing the promotion of healthy lifestyles within the prison environment. Integrating regular assessments of prisoners’ physical activity levels upon entering the penal system and while serving their sentences can guide specific, personalized interventions to promote more active habits. Research in this context can inform the development of specific physical activity programs that help incarcerated people cope with stress and promote active lifestyles. Strategies such as implementing regular physical activity, sports, and outdoor exercise can be beneficial in reducing a sedentary lifestyle and improving overall physical health [11]. The urgent need for additional research is evident, aiming to better understand the effects of physical activity in prisons and design effective interventions to improve the health of inmates. Promoting massive health education, along with physical activity, emerges as an integral strategy to improve the overall health of the prison population, recognizing these factors as essential components for a comprehensive care approach [49,50]. Furthermore, the COVID-19 pandemic deteriorated the overall health of people in prison, in terms of both mental and physical health [51]. Finally, government structures must implement programs aimed at promoting physical activity and adopting healthy eating habits as an essential part of health initiatives for prisoners. These holistic approaches not only aim to safeguard physical health but also contribute to the effective management of overall well-being. In this vein, there is a high need for the provision of adequate facilities to promote physical activity in the prison context.

## 5. Conclusions

Most study participants failed to meet the global recommendations for physical activity. Age and sitting time were associated with physical inactivity, which raises the risk of future health problems. In sum, the results of this research highlight the importance of promoting physical activity and health in prison environments. We highlighted gaps in the implementation of effective practices, despite existing legal guidelines. The relationship between physical inactivity, precarious conditions, and health problems, such as hypertension, demands urgent action to improve the living conditions and well-being of the Brazilian prison population. Given the aging of the incarcerated population and high rates of chronic illness, we highlight the need for integrated policies that address not only physical activity but also eating habits and adequate access to healthcare. Addressing these challenges will significantly contribute to promoting the health and social reintegration of these individuals.

## Figures and Tables

**Table 1:** Participant characteristics by physical activity practice (N = 216).

Variables			Active Physical Activity (WHO Recommendation)				*p*
			No (n = 139; 64.3%)		Yes (n = 77; 35.7%)		
**Sociodemographic**							
**Age group**	n	%	n	%	N	%	
18 to 29	115	53.2	65	46.8	50	64.9	0.034 *
30 to 44	79	36.6	57	41.0	22	28.6	
45 to 59	22	10.2	17	12.2	5	6.5	
**Race**							
White	83	38.4	51	36.7	32	41.6	0.577
Black or African American	133	61.6	88	63.3	45	58.4	
**Years of study**							
<8	131	60.6	86	61.9	45	58.4	0.727
≥8	85	39.4	53	38.1	32	41.6	
**Have a partner**							
No	63	29.2	41	29.5	22	28.6	0.999
Yes	153	70.8	98	70.5	55	71.4	
**Have children**							
No	75	34.7	44	31.7	31	40.3	0.261
Yes	141	65.3	95	68.3	46	59.7	
**Living conditions before incarceration**							
**Housing condition**	n	%	n	%	n	%	
Own home	130	60.2	84	60.4	46	59.7	0.999
Rented	86	39.8	55	39.6	31	40.3	
**Paid work**							
No	24	11.1	19	13.7	5	6.5	0.167
yes	192	88.9	120	86.3	72	93.5	
**Financial income †**							
<1 minimum wage	26	12.0	19	13.7	7	9.1	0.440
≥1 minimum wage	190	88.0	120	86.3	70	90.9	
**Information about current incarceration**							
**Previous incarceration history**	n	%	n	%	n	%	
No	57	26.4	35	25.2	22	28.6	0.704
Yes	159	73.6	104	74.8	55	71.4	
**Time of incarceration**							
<1 year	156	72.2	99	71.2	57	74.0	0.778
≥1 year	60	27.8	40	28.8	20	26.0	
**Work inside prison**							
No	179	82.9	114	82.0	65	84.4	0.795
Yes	37	17.1	25	18.0	12	15.6	
**Have visitors in last month**							
No	70	32.4	47	33.8	23	29.9	0.659
Yes	146	67.6	92	66.2	54	70.1	
**Health and lifestyle information**							
**Body mass index**	n	%	n	%	n	%	
Eutrophic	108	50.2	67	48.6	41	53.2	0.215
Overweight	82	38.1	51	37.0	31	40.3	
Obesity	25	11.6	20	14.5	5	6.5	
**Blood pressure**							
Normal (<129/84 mmHg)	137	63.4	88	63.3	49	63.6	0.997
Limit/high (>130/85 mmHg)	79	36.6	51	36.7	28	36.4	
**Food intake**							
Healthy	100	46.3	71	51.1	29	37.7	0.080
Unhealthy	116	53.7	68	48.9	48	62.3	
**Sitting time (hours)**							0.001 *
Up to 12	57	26.5	24	17.3	33	43.4	
More than 12	158	73.5	115	82.7	43	56.6	

† Based on the value of BRL 998.00 (minimum wage in 2019); * Statistically significant; test of significance = chi-square test.

**Table 2:** Multivariable logistic regression for factors associated with lower physical activity among inmates.

Variables and Categories	Univariate Analysis					Final Model				
	Estimates	Crude OR (95% CI)			*p*	Estimates	AOR (95% CI)			*p*
**Age (years)**	−0.0453	0.96 (0.96–0.99)			0.008 *	−0.0483	0.95 (0.92–0.99)			0.010 *
**Race**										
White	Reference	-			-	-	-	-	-	-
Black/African descent	−0.2046	0.81 (0.46–1.44)			0.481	-	-	-	-	-
**Years of study**										
<8	Reference		-		-	-	-	-	-	-
≥8	0.1430	1.15 (0.65–2.04)			0.621	-	-	-	-	-
**Have a partner**										
No	Reference		-		-	-	-	-	-	-
Yes	0.0449	1.05 (0.57–1.93)			0.886	-	-	-	-	-
**Children**										
No	Reference		-		-	-	-	-	-	-
Yes	−0.3750	0.69 (0.39–1.23)			0.204	-	-	-	-	-
**Housing condition**										
Own home	Reference		-		-	-	-	-	-	-
Rented	0.0288	1.03 (0.58–1.82)			0.921	-	-	-	-	-
**Paid work**										
No	Reference	-			-	-	-	-	-	-
yes	0.8242	2.28 (0.82–6.37)			0.116	-	-	-	-	-
**Financial income †**										
<1 minimum wage	Reference		-			-	-	-	-	-
≥1 minimum wage	0.4595	1.58 (0.63–3.95)			0.325	-	-	-	-	-
**Previous incarceration history**										
No	Reference		-			-	-	-	-	-
Yes	−0.1728	0.84 (0.45–1.57)			0.588	-	-	-	-	-
**Time of incarceration**										
<1 year	Reference		-			-	-	-	-	-
≥1 year	0.0004	1.00 (0.99–1.01)			0.487	-	-	-	-	-
**Work inside prison**										
No	Reference		-			-	-	-	-	-
Yes	−0.1722	0.84 (0.4–1.79)			0.043 *	1.0611	2.89 (0.93–8.93)			0.065
**Have visitors**										
No	Reference		-							
Yes	0.1818	1.2 (0.66–2.19)			0.553	-	-	-	-	-
**Sitting time (hours)**	0.3152	0.73 (0.64–0.83)			<0.001 *	−0.2995	0.74 (0.65–0.85)			<0.001 *
**Body mass index**										
Eutrophic	Reference		-							
Overweight	−0.0067	0.99 (0.55–1.8)			0.982	-	-	-	-	-
Obesity	−0.8952	0.41 (0.14–1.17)			0.096	-	-	-	-	-
**Blood pressure**										
Normal (<129/84 mmHg)	Reference		-			-	-	-	-	-
Limit/high (>130/85 mmHg)	−0.0141	0.99 (0.55–1.76)			0.962	-	-	-	-	-
**Food intake**										
Healthy	Reference		-			-	-	-	-	-
Unhealthy	−0.5471	0.58 (0.33–1.02)			0.049 *	−0.4634	0.63 (0.34–1.18)			0.149

† based on the value of BRL 998.00 [minimum wage in 2019]; AOR = adjusted odds ratio; * = statistically significant; - did not appear in the final step of multivariate logistic regression.

## Data Availability

All data generated or analyzed during this study are included in this article. This article is based on the first author’s master’s dissertation in Nursing at the State University of Maringá, Brazil.
